# Supplementary material for: Application of a Multilayer Perceptron Artificial Neural Network for the Prediction and Optimization of the Andrographolide Content in Andrographis paniculata
Source: Molecules. 2022 Apr 26;27(9):2765. doi: 10.3390/molecules27092765 (PMC9105688; doi:10.3390/molecules27092765)
Supplement: Supplementary file 1 [file molecules-27-02765-s001.zip › molecules-1690191-supplementary.pdf]

Supplementary materials

Table S1. Actual and predicted Andrographolide content of training dataset.

Acc. No.	Target	Output	Absolute Error	Square of Absolute Error	Absolute Percentage Error
AP-1	1.335	1.392	0.058	0.003	4.331
AP-10	1.303	1.351	0.048	0.002	3.705
AP-11	1.860	1.925	0.065	0.004	3.517
AP-13	1.926	1.845	0.082	0.007	4.239
AP-14	2.865	2.758	0.107	0.011	3.736
AP-20	1.466	1.515	0.049	0.002	3.354
AP-23	0.975	0.896	0.078	0.006	8.050
AP-24	2.812	3.026	0.214	0.046	7.624
AP-26	4.412	4.264	0.148	0.022	3.364
AP-34	2.719	3.065	0.347	0.120	12.749
AP-35	0.646	0.715	0.069	0.005	10.738
AP-37	1.906	1.892	0.014	0.000	0.726
AP-38	3.555	3.915	0.360	0.129	10.118
AP-39	3.545	3.814	0.270	0.073	7.607
AP-21	0.778	0.825	0.047	0.002	6.054
AP-44	1.467	1.622	0.155	0.024	10.543
AP-46	2.617	2.755	0.139	0.019	5.298
AP-12	2.569	2.921	0.352	0.124	13.712
AP-55	1.236	1.400	0.164	0.027	13.272
AP-56	2.464	2.571	0.106	0.011	4.321
AP-60	2.758	2.569	0.189	0.036	6.860
AP-2	2.455	2.817	0.362	0.131	14.749
AP-3	1.028	1.162	0.134	0.018	13.015
AP-4	1.694	1.680	0.014	0.000	0.844
AP-56	1.952	1.846	0.106	0.011	5.438
AP-73	2.928	2.937	0.009	0.000	0.292
AP-74	1.011	1.052	0.041	0.002	4.038
AP-61	1.543	1.850	0.307	0.094	19.876
AP-62	1.386	1.366	0.020	0.000	1.464
AP-63	1.778	1.547	0.231	0.053	12.986
AP-64	2.501	2.301	0.200	0.040	7.994
AP-65	1.281	1.308	0.027	0.001	2.135
AP-66	0.862	0.712	0.150	0.023	17.436
AP-72	2.945	2.968	0.023	0.001	0.778
AP-76	1.049	1.127	0.078	0.006	7.443
AP-77	1.645	1.770	0.125	0.016	7.630

AP-78	1.632	1.456	0.176	0.031	10.796
AP-79	1.218	1.372	0.154	0.024	12.674
AP-48	1.962	1.931	0.032	0.001	1.621
AP-18	1.502	1.353	0.149	0.022	9.911
AP-19	1.739	1.557	0.182	0.033	10.457
AP-82	0.849	0.832	0.018	0.000	2.084
AP-40	0.730	0.812	0.082	0.007	11.239
AP-83	0.756	0.833	0.077	0.006	10.230
AP-86	2.251	2.358	0.106	0.011	4.718
AP-87	1.173	1.076	0.097	0.009	8.256
AP-89	1.682	1.554	0.128	0.016	7.607
AP-91	1.145	1.297	0.152	0.023	13.308
AP-27	1.304	1.224	0.081	0.006	6.177
AP-28	0.769	0.804	0.034	0.001	4.453
AP-29	3.759	3.349	0.409	0.168	10.893
AP-30	5.230	5.157	0.073	0.005	1.389
AP-31	5.394	4.884	0.510	0.260	9.456
AP-33	3.743	3.391	0.352	0.124	9.394
AP-6	3.099	3.272	0.173	0.030	5.572
AP-8	2.832	2.466	0.366	0.134	12.922
AP-51	2.678	2.328	0.350	0.122	13.065
AP-52	3.760	3.460	0.300	0.090	7.983
AP-53	3.680	3.176	0.504	0.254	13.702
AP-67	3.523	3.461	0.062	0.004	1.763
AP-68	3.544	3.210	0.334	0.112	9.437
AP-69	3.515	3.346	0.169	0.029	4.819
AP-42	3.918	3.781	0.136	0.019	3.480
AP-43	2.943	2.568	0.375	0.141	12.748
AP-85	2.030	2.292	0.262	0.069	12.902
AP-92	1.667	1.721	0.054	0.003	3.249
AP-123	2.248	2.523	0.275	0.076	12.242
AP-93	1.770	1.634	0.136	0.019	7.696
AP-127	0.772	0.823	0.050	0.003	6.523
AP-130	0.280	0.302	0.022	0.000	7.929
AP-131	0.648	0.587	0.062	0.004	9.534
AP-135	0.802	0.724	0.078	0.006	9.758
AP-136	0.988	0.879	0.108	0.012	10.977
AP-137	0.319	0.340	0.021	0.000	6.500
AP-94	1.350	1.370	0.020	0.000	1.496
AP-143	0.358	0.382	0.024	0.001	6.752

AP-116	0.411	0.458	0.047	0.002	11.317
AP-140	0.463	0.506	0.043	0.002	9.204
AP-124	1.231	1.099	0.132	0.017	10.688
AP-132	0.802	0.837	0.035	0.001	4.340
AP-144	0.910	0.812	0.097	0.009	10.706
AP-96	1.486	1.499	0.014	0.000	0.925
AP-117	0.774	0.689	0.084	0.007	10.900
AP-145	2.028	1.968	0.060	0.004	2.950
AP-147	1.352	1.185	0.167	0.028	12.325
AP-149	0.338	0.357	0.019	0.000	5.509
AP-118	0.669	0.616	0.053	0.003	7.966
AP-98	0.355	0.371	0.016	0.000	4.479
AP-146	2.483	2.296	0.187	0.035	7.538
AP-99	2.693	2.503	0.190	0.036	7.073
AP-100	0.284	0.314	0.030	0.001	10.621
AP-150	1.992	2.045	0.053	0.003	2.682
AP-102	1.990	2.056	0.067	0.004	3.348
AP-104	1.499	1.351	0.148	0.022	9.859
AP-105	1.555	1.429	0.126	0.016	8.122
AP-106	1.882	1.694	0.188	0.035	9.995
AP-119	1.906	1.695	0.211	0.045	11.070
AP-142	2.013	1.898	0.115	0.013	5.696
AP-109	1.998	1.896	0.102	0.010	5.098
AP-121	1.923	1.789	0.134	0.018	6.971
AP-110	1.580	1.449	0.131	0.017	8.307
AP-111	1.661	1.559	0.102	0.010	6.159
AP-148	2.603	2.891	0.288	0.083	11.067
AP-122	1.952	2.082	0.130	0.017	6.664
AP-138	0.475	0.510	0.035	0.001	7.344
AP-133	3.582	3.437	0.146	0.021	4.062
			Mean	0.0322	7.6295
			RMSE	0.1793	

Table S2. Actual and predicted Andrographolide content of testing dataset.

Acc. No.	Target	Output	Absolute Error	Square of Absolute Error	Absolute Percentage Error
AP-15	1.094	1.149	0.055	0.003	5.032
AP-25	2.585	2.981	0.396	0.157	15.306
AP-45	3.643	3.964	0.321	0.103	8.819
AP-59	2.805	2.521	0.284	0.081	10.138

AP-71	4.664	4.622	0.042	0.002	0.903
AP-75	3.047	2.671	0.376	0.141	12.344
AP-17	4.707	4.293	0.414	0.171	8.797
AP-80	0.854	0.874	0.020	0.000	2.303
AP-41	4.222	4.170	0.052	0.003	1.234
AP-88	1.733	1.533	0.200	0.040	11.553
AP-7	4.927	4.349	0.577	0.333	11.718
AP-54	3.022	3.359	0.337	0.113	11.145
AP-84	2.964	2.922	0.042	0.002	1.420
AP-113	1.024	1.059	0.035	0.001	3.427
AP-115	0.600	0.562	0.038	0.001	6.335
AP-95	1.453	1.226	0.227	0.051	15.606
AP-125	0.799	0.748	0.052	0.003	6.470
AP-141	1.909	1.722	0.187	0.035	9.774
AP-129	2.250	2.506	0.257	0.066	11.406
AP-126	2.627	2.472	0.155	0.024	5.894
AP-112	1.932	2.192	0.260	0.068	13.458
AP-134	1.960	2.023	0.063	0.004	3.235
			Average	0.064	7.907
			RMSE	0.253	

Table S3. Actual and predicted Andrographolide content of validation dataset.

Acc. No.	Target	Output	Absolute Error	Square of Absolute Error	Absolute Percentage Error
AP-94	0.676	0.662	0.014	0.000	2.063
AP-22	1.822	1.603	0.219	0.048	12.009
AP-47	1.661	1.815	0.154	0.024	9.285
AP-57	2.460	2.743	0.284	0.080	11.527
AP-58	2.824	2.573	0.251	0.063	8.877
AP-16	2.990	2.731	0.260	0.067	8.679
AP-49	1.042	1.191	0.149	0.022	14.345
AP-90	1.386	1.552	0.166	0.028	12.009
AP-32	3.972	3.510	0.462	0.213	11.624
AP-5	4.288	4.141	0.148	0.022	3.442
AP-9	5.446	4.891	0.555	0.308	10.195
AP-81	4.368	4.034	0.334	0.111	7.640
AP-50	2.744	2.589	0.156	0.024	5.667
AP-128	0.650	0.642	0.008	0.000	1.254
AP-114	0.573	0.613	0.040	0.002	7.039
AP-139	0.338	0.351	0.013	0.000	3.947

AP-97	0.383	0.351	0.033	0.001	8.527
AP-101	0.350	0.364	0.014	0.000	4.035
AP-103	1.444	1.285	0.159	0.025	11.010
AP-107	1.536	1.524	0.012	0.000	0.761
AP-108	0.661	0.558	0.103	0.011	15.633
AP-120	1.857	1.804	0.053	0.003	2.832
			Mean	0.048	7.836
			RMSE	0.219	

Table S4. Geographic locations and habitats characteristics of *Andrographis paniculata* populations.

Serial No.	Acc. No	Place	State & District	Latitude and Longitude	Year of sample collection	Voucher No.
1	AP-1	Ekamra garden	Odisha, Khordha	20° 16' 19.20" N, 85° 49' 58.80" E	2019	1098/CBT
2	AP-2	Barunei road	Odisha, Khordha	20° 10' 15.60" N, 85° 38' 60.00" E	2019	1100/CBT
3	AP-3	Barunei pitha	Odisha, Khordha	20° 09' 39.60" N, 85° 38' 52.80" E	2019	1101/CBT
4	AP-4	Baibhava vihar	Odisha, Khordha	20° 10' 55.20" N, 85° 39' 18.00" E	2019	1102/CBT
5	AP-5	Mukudaprasad	Odisha, Khordha	20° 10' 12.00" N, 85° 38' 20.40" E	2019	1103/CBT
6	AP-6	Barunei pitha	Odisha, Khordha	20° 09' 39.60" N, 85° 38' 52.80" E	2019	1104/CBT
7	AP-7	Barunei pitha	Odisha, Khordha	20° 09' 32.40" N, 85° 38' 42.00" E	2019	1105/CBT
8	AP-8	Gada Khordha	Odisha, Khordha	20° 09' 46.80" N, 85° 37' 48.00" E	2019	1106/CBT
9	AP-9	Gada Khordha	Odisha, Khordha	20° 09' 36.00" N, 85° 37' 44.40" E	2019	1108/CBT
10	AP-10	Baliapala	Odisha, Balasore	21° 39' 14.40" N, 87° 16' 58.80" E	2019	1126/CBT
11	AP-11	Nilagiri	Odisha, Balasore	21° 27' 39.60" N, 86° 45' 21.60" E	2019	1127/CBT
12	AP-12	Jamalpur	Odisha, Balasore	21° 29' 33.50" N, 86° 56' 00.50" E	2019	1128/CBT
13	AP-13	Ambapua	Odisha, Ganjam	19° 19' 01.90" N, 84° 50' 50.30" E	2019	1129/CBT
14	AP-14	Kholikot college	Odisha, Ganjam	19° 18' 28.00" N, 84° 47' 39.40" E	2019	1130/CBT
15	AP-15	Berhampur University	Odisha, Ganjam	19° 17' 42.00" N, 84° 52' 48.00" E	2019	1131/CBT
16	AP-16	Nirmalajhara	Odisha, Ganjam	19° 35' 45.60" N, 85° 03' 39.60" E	2019	1133/CBT
17	AP-17	Bharasa	Odisha, Ganjam	19° 36' 25.200" N, 85° 3' 21.60" E	2019	1134/CBT
18	AP-18	Tamana	Odisha, Ganjam	19° 13' 58.80" N, 84° 40' 58.80" E	2019	1135/CBT
19	AP-19	Taptapani	Odisha, Ganjam	19° 28' 51.60" N, 84° 25' 26.40" E	2019	1136/CBT
20	AP-20	Naraja	Odisha, cuttack	20° 28' 12.00" N, 85° 46' 40.80" E	2019	1113/CBT
21	AP-21	Radhakishorepur	Odisha, Cuttack	20° 33' 25.20" N, 85° 45' 25.20" E	2019	1114/CBT
22	AP-22	Chodwar	Odisha, Cuttack	20° 31' 26.40" N, 85° 54' 10.80" E	2019	1115/CBT
23	AP-23	Paralakhemundi	Odisha, Gajapati	18° 46' 40.80" N, 84° 06' 32.40" E	2019	1137/CBT
24	AP-24	Madaba	Odisha, Gajapati	18° 57' 58.10" N, 84° 20' 57.80" E	2019	1138/CBT
25	AP-25	Budisila	Odisha, Gajapati	19° 07' 55.20" N, 84° 07' 51.60" E	2019	1139/CBT
26	AP-26	Rayagada	Odisha, Gajapati	18° 59' 16.80" N, 84° 08' 02.40" E	2019	1140/CBT
27	AP-27	Barghala	Odisha, Gajapati	18° 52' 40.80" N, 84° 11' 45.60" E	2019	1141/CBT
28	AP-28	Hatapada	Odisha, Gajapati	18° 57' 46.80" N, 84° 09' 50.40" E	2019	1142/CBT

29	AP-29	Mahendragiri	Odisha, Gajapati	18° 59' 49.20" N, 84° 07' 40.80" E	2019	1143/CBT
30	AP-30	Budisila	Odisha, Gajapati	19° 07' 51.60" N, 84° 07' 55.20" E	2019	1144/CBT
31	AP-31	Budisila	Odisha, Gajapati	19° 07' 48.00" N, 84° 07' 55.20" E	2019	1145/CBT
32	AP-32	Karadasigh	Odisha, Gajapati	18° 55' 26.40" N, 84° 12' 25.20" E	2019	1146/CBT
33	AP-33	Alluri Nagar	Odisha, Gajapati	18° 48' 46.80" N, 84° 08' 38.40" E	2019	1147/CBT
34	AP-34	Nimapada	Odisha, Puri	20° 03' 21.60" N, 86° 0.0' 14.40" E	2019	1109/CBT
35	AP-35	Balisahi	Odisha, Puri	19° 57' 57.60" N, 86° 01' 33.60" E	2019	1110/CBT
36	AP-36	Khalakata patna	Odisha, Puri	19° 53' 14.60" N, 86° 05' 40.50" E	2019	1111/CBT
37	AP-37	Banpur	Odisha, Puri	20° 05' 52.80" N, 85° 53' 38.40" E	2019	1112/CBT
38	AP-38	Jharabandha	Odisha, Dhenkanala	20° 32' 24.00" N, 85° 34' 51.60" E	2019	1117/CBT
39	AP-39	Deagaon	Odisha, Dhenkanala	20° 41' 49.90" N, 85° 44' 50.60" E	2019	1119/CBT
40	AP-40	Gandanali	Odisha, Dhenkanala	20° 35' 52.80" N, 85° 21' 03.60" E	2019	1120/CBT
41	AP-41	Baunsapokhari	Odisha, Dhenkanala	20° 39' 50.40" N, 85° 10' 44.40" E	2019	1121/CBT
42	AP-42	Mahulakhali	Odisha, Dhankanal	20° 41' 13.20" N, 85° 44' 24.00" E	2019	1122/CBT
43	AP-43	Kapilash	Odisha, Dhankanal	20° 41' 13.20" N, 85° 45' 25.20" E	2019	1123/CBT
44	AP-44	Chitrada	Odisha, Mayurbhanj	21° 50' 58.50" N, 86° 56' 41.60" E	2019	1155/CBT
45	AP-45	Betnoti	Odisha, Mayurbhanj	21° 44' 27.60" N, 86° 50' 42.00" E	2019	1156/CBT
46	AP-46	Baisingha	Odisha, Mayurbhanj	21° 38' 47.40" N, 86° 54' 53.28" E	2019	1157/CBT
47	AP-47	Betnosia	Odisha, Mayurbhanj	21° 44' 23.60" N, 86° 50' 35.50" E	2019	1158/CBT
48	AP-48	Shankarpur	Odisha, Mayurbhanj	22° 00' 57.60" N, 85° 42' 32.40" E	2019	1159/CBT
49	AP-49	Phulkong forest	Odisha, Mayurbhanj	22° 01' 12.00" N, 85° 45' 39.60" E	2019	1160/CBT
50	AP-50	Similipala road	Odisha, Mayurbhanj	21° 53' 16.80" N, 86° 11' 38.40" E	2019	1149/CBT
51	AP-51	Jashipur	Odisha, Mayurbhanj	21° 53' 27.60" N, 86° 11' 49.20" E	2019	1150/CBT
52	AP-52	Similipala road	Odisha, Mayurbhanj	21° 54' 07.20" N, 86° 11' 24.00" E	2019	1151/CBT
53	AP-53	Baman Ghati	Odisha, Mayurbhanj	22° 10' 08.40" N, 86° 29' 38.40" E	2019	1153/CBT
54	AP-54	Baman Ghati	Odisha, Mayurbhanj	22° 09' 54.00" N, 86° 31' 15.60" E	2019	1154/CBT
55	AP-55	Khandapada	Odisha, Nayagargh	20° 12' 36.00" N, 85° 08' 24.00" E	2019	1164/CBT
56	AP-56	Sampada	Odisha, Nayagargh	20° 12' 36.00" N, 84° 58' 26.40" E	2019	1165/CBT
57	AP-57	Gania	Odisha, Nayagargh	20° 12' 36.00" N, 84° 58' 26.40" E	2019	1166/CBT
58	AP-58	Pratap prasad	Odisha, Nayagargh	20° 07' 51.60" N, 85° 08' 31.20" E	2019	1167/CBT
59	AP-59	Palli sasan	Odisha, Nayagargh	20° 04' 58.80" N, 85° 06' 18.00" E	2019	1168/CBT
60	AP-60	Ratanpur	Odisha, Nayagargh	20° 07' 55.20" N, 85° 06' 36.00" E	2019	1169/CBT
61	AP-61	Kakalama	Odisha, Nayagargh	20° 04' 30.00" N, 85° 15' 18.00" E	2019	1170/CBT
62	AP-62	Itamati	Odisha, Nayagargh	20° 0.0' 10.80" N, 85° 22' 19.20" E	2019	1171/CBT
63	AP-63	Odagaon	Odisha, Nayagargh	20° .00' 14.40" N, 84° 59' 34.80" E	2019	1172/CBT
64	AP-64	Krushnaprasad)	Odisha, Nayagargh	20° 08' 19.64" N, 85° 08' 41.28" E	2019	1173/CBT
65	AP-65	Sarankula	Odisha, Nayagargh	20° 02' 13.20" N, 85° 04' 40.80" E	2019	1174/CBT
66	AP-66	Banigochha	Odisha, Nayagargh	20° 24' 01.80" N, 84° 35' 11.04" E	2019	1175/CBT
67	AP-67	Godi Alli	Odisha, Nayagargh	20° 07' 58.80" N, 85° 08' 24.00" E	2019	1176/CBT
68	AP-68	Godi Alli	Odisha, Nayagargh	20° 06' 43.20" N, 85° 05' 13.20" E	2019	1177/CBT
69	AP-69	Baigunia	Odisha, Nayagargh	20° 15' 28.80" N, 85° 12' 39.60" E	2019	1178/CBT
70	AP-70	Korada ghati	Odisha,Kandhamala	19° 57' 50.40" N, 84° 19' 37.20" E	2019	1183/CBT
71	AP-71	Raikia	Odisha,Kandhamala	19° 58' 51.60" N, 84° 14' 24.00" E	2019	1184/CBT
72	AP-72	Phiringia	Odisha, Kandhamala	20° 10' 55.20" N, 84° 04' 44.40" E	2019	1188/CBT
73	AP-73	Nuabarada	Odisha, Jajpur	20° 47' 38.40" N, 86° 02' 60.00" E	2019	1193/CBT

74	AP-74	Panikoili	Odisha, Jajpur	20° 54' 43.20" N, 86° 13' 40.80" E	2019	1194/CBT
75	AP-75	Udayagiri	Odisha, Jajpur	20° 37' 37.20" N, 86° 14' 42.00" E	2019	1195/CBT
76	AP-76	Ranipathara	Odisha, Baudh (Phulbani)	20° 29' 20.40" N, 84° 15' 28.80" E	2019	1190/CBT
77	AP-77	Phurlijharan	Odisha, Kalahandi	19° 46' 54.22" N, 83° 06' 49.90" E	2019	1191/CBT
78	AP-78	Jagasahi	Odisha, Kalahandi	19°45' 00.70" N, 83° 11' 45.12" E	2019	1192/CBT
79	AP-79	Magurgadia	Odisha, keonjhar	21° 38' 45.60" N, 85° 36' 25.20" E	2019	1196/CBT
80	AP-80	Judia ghati	Odisha, keonjhar	21° 37' 44.40" N, 85° 34' 12.00" E	2019	1197/CBT
81	AP-81	Hadagargh	Odisha, Keonjhar	21° 17' 21.12" N, 86° 19' 11.64" E	2019	1198/CBT
82	AP-82	Tirtol	Odisha, Jagatsighpur	20° 20' 21.08" N, 86° 05' 52.04" E	2019	1124/CBT
83	AP-83	Chandanpur	Odisha, Angul	20° 46' 04.80" N, 84° 58' 48.00" E	2019	1179/CBT
84	AP-84	Bedashasana	Odisha, Angul	20° 42' 30.16" N, 85° 07' 58.80" E	2019	1180/CBT
85	AP-85	Tarava	Odisha, Angul	20° 42' 05.11" N, 84° 51' 13.89" E	2019	1181/CBT
86	AP-86	Laxmi Dungri	Odisha, Sambalpur	21° 29' 56.40" N, 83° 55' 30.00" E	2019	1161/CBT
87	AP-87	Ashok Niwas	Odisha, Sambalpur	21° 30' 43.20" N, 83° 51' 10.80" E	2019	1162/CBT
88	AP-88	Sanapalli	Odisha, Sambalpur	21° 23' 13.20" N, 83° 56' 20.40" E	2019	1163/CBT
89	AP-89	Therubali	Odisha, Rayagada	19° 17' 56.40" N, 83° 26' 27.60" E	2019	1199/CBT
90	AP-90	Kalyansighpur	Odisha, Rayagada	19° 10' 48.00" N, 83° 25' 01.20" E	2019	1200/CBT
91	AP-91	Padampur	Odisha, Rayagada	19° 14' 35.73" N, 83° 49' 33.96" E	2019	1201/CBT
92	AP-92	Behela	West Bengal, South 24 Parganas	22° 29' 52.08" N, 88° 18' 36.00" E	2019	1202/CBT
93	AP-93	Kakadwip	West Bengal, South 24 Parganas	21° 52' 33.60" N, 88° 11' 07.08" E	2019	1203/CBT
94	AP-94	Diamond harbour	West Bengal, South 24 Parganas	22° 11' 52.80" N, 88° 12' 07.20" E	2019	1204/CBT
95	AP-95	Kulpi	West Bengal, South 24 Parganas	22° 04' 51.60" N, 88° 14' 38.40" E	2019	1205/CBT
96	AP-96	Kakadwip	West Bengal, South 24 Parganas	21° 52' 33.60"N, 88° 11' 07.08" E	2019	1206/CBT
97	AP-97	Kakadwip	West Bengal, South 24 Parganas	21° 52' 33.60"N, 88° 11' 07.08" E	2019	1207/CBT
98	AP-98	Laxmikantapur	West Bengal, South 24 Parganas	22° 06' 35.64" N, 88° 19' 15.24" E	2019	1208/CBT
99	AP-99	Amtala	West Bengal, South 24 Parganas	22° 22' 00.84" N, 88° 16' 39.72" E	2019	1209/CBT
100	AP-100	Patharapratima	West Bengal, South 24 Parganas	21° 47' 31.20" N, 88° 21' 18.00" E	2019	1210/CBT
101	AP-101	Baharu	West Bengal, South 24 Parganas	22° 12' 14.40" N, 88° 25' 41.88" E	2019	1211/CBT
102	AP-102	Raidighi	West Bengal, South 24 Parganas	22° 00' 04.32" N, 88° 26' 07.44" E	2019	1212/CBT
103	AP-103	Thakurpukur	West Bengal, South 24 Parganas	22° 27' 39.60" N, 88° 18' 21.60" E	2019	1213/CBT
104	AP-104	Garia	West Bengal, South 24 Parganas	22° 27' 57.60" N, 88° 23' 34.08" E	2019	1214/CBT
105	AP-105	Narayanpur	West Bengal, South 24 Parganas	21° 46′ 15.00″ N, 88°15′ 04.00″ E	2019	1215/CBT
106	AP-106	Nischintapur	West Bengal, South 24 Parganas	21° 59' 24.00" N, 88° 12' 49.32" E	2019	1216/CBT
107	AP-107	Dholahat	West Bengal, South 24 Parganas	22° 01' 22.44" N, 88° 18' 01.80" E	2019	1217/CBT
108	AP-108	Kalyanpur	West Bengal, South 24 Parganas	22° 21' 02.88" N, 88° 25' 01.56" E	2019	1218/CBT
109	AP-109	Kashinagar	West Bengal, South 24 Parganas	22° 05' 21.48" N, 88° 25' 28.56" E	2019	1219/CBT
110	AP-110	Karanjali	West Bengal, South 24 Parganas	22° 01' 44.40" N, 88° 14' 52.80" E	2019	1220/CBT
111	AP-111	Jaynagar	West Bengal, South 24 Parganas	22° 10' 26.40" N, 88° 25' 22.80" E	2019	1221/CBT
112	AP-112	Baruipur	West Bengal, South 24 Parganas	22° 21' 32.40" N, 88° 25' 55.20" E	2019	1222/CBT
113	AP-113	Barrackpore	West Bengal, North 24 Parganas	22° 46' 02.64" N, 88° 23' 17.88" E	2019	1223/CBT
114	AP-114	Titagargh	West Bengal, North 24 Parganas	22° 44' 17.88" N, 88° 22' 25.32" E	2019	1224/CBT
115	AP-115	Barasat	West Bengal, North 24 Parganas	22° 43' 29.28" N, 88° 28' 44.04" E	2019	1226/CBT
116	AP-116	Salt Lake	West Bengal, North 24 Parganas	22° 34' 46.92" N, 88° 24' 51.48" E	2019	1227/CBT
117	AP-117	Bandipur	West Bengal, North 24 Parganas	22° 43' 47.64" N, 88° 23' 42.72" E	2019	1228/CBT
118	AP-118	Rahara	West Bengal, North 24 Parganas	22° 43' 26.40" N, 88° 23' 12.48" E	2019	1229/CBT

119	AP-119	Habra	West Bengal, North 24 Parganas	22° 51' 19.80" N, 88° 44' 38.40" E	2019	1230/CBT
120	AP-120	Sworupnagar	West Bengal, North 24 Parganas	22° 48' 39.60" N, 88° 51' 21.60" E	2019	1231/CBT
121	AP-121	Madhyamgram	West Bengal, North 24 Parganas	22° 41' 32.64" N, 88° 27' 55.08" E	2019	1232/CBT
122	AP-122	Naihati	West Bengal, North 24 Parganas	22° 53' 20.40" N, 88° 25' 19.20" E	2019	1233/CBT
123	AP-123	Kulera,	West Bengal, Birbhum	23° 54' 28.80" N, 87° 32' 52.80" E	2019	1250/CBT
124	AP-124	RK mission	West Bengal, Birbham	23° 50' 24.72" N, 23° 50' 24.72" E	2019	1251/CBT
125	AP-125	Suri	West Bengal, Birbhum	23° 54' 14.40" N, 87° 31' 26.40" E	2019	1252/CBT
126	AP-126	Shantiniketan (Bolpur)	West Bengal, Birbhum	23° 40' 37.20" N, 87° 41' 06.00" E	2019	1253/CBT
127	AP-127	Kaliagangh	West Bengal, Uttar Dinajpur	25° 38' 06.00" N, 88° 19' 40.80" E	2019	1262/CBT
128	AP-128	Jangipara	West Bengal, Hooghly	22° 44' 10.68" N, 88° 03' 29.88" E	2019	1240/CBT
129	AP-129	Serampore	West Bengal, Hooghly	22° 44' 52.80" N, 88° 20' 16.80" E	2019	1241/CBT
130	AP-130	Salboni	West Bengal, Paschim Medinipur	22° 38' 31.20" N, 87° 19' 08.40" E	2019	1239/CBT
131	AP-131	Marishda	West Bengal, Purba Medinipur	21° 51' 01.80" N, 87° 44' 39.48" E	2019	1235/CBT
132	AP-132	Tamluk	West Bengal, Purba Medinipur	22° 17' 08.88" N, 22° 17' 08.88" E	2019	1236/CBT
133	AP-133	Haldia	West Bengal, Purba Medinipur	22° 03' 45.72" N, 88° 04' 59.88" E	2019	1237/CBT
134	AP-134	Ramnagar	West Bengal, Purba Medinipur	21° 40' 40.80" N, 87° 33' 46.80 "E	2019	1238/CBT
135	AP-135	Bankura	West Bengal, Bankura	23° 13' 52.68" N, 87° 04' 42.24" E	2019	1245/CBT
136	AP-136	Bankura	West Bengal, Bankura	23° 15' 18.72" N, 87° 05' 38.40" E	2019	1246/CBT
137	AP-137	Bankura	West Bengal, Bankura	23° 13' 52.68" N, 87° 04' 42.24" E	2019	1247/CBT
138	AP-138	Khatra	West Bengal,Bankura	22° 58' 23.16" N, 86° 51' 16.92" E	2019	1248/CBT
139	AP-139	Nabadwip	West Bengal, nadia	23° 24' 10.80" N, 23° 24' 10.80" E	2019	1253/CBT
140	AP-140	Kalyani	West Bengal, Nadia	22° 58' 26.40" N, 88° 25' 58.80" E	2019	1254/CBT
141	AP-141	Debagram	West Bengal, Nadia	23° 41' 16.80" N, 88° 18' 32.40" E	2019	1255/CBT
142	AP-142	Krishnanagar	West Bengal, Nadia	23° 24' 03.60" N, 88° 30' 07.20" E	2019	1257/CBT
143	AP-143	Siliguri	West Bengal, Darjeeling	26° 43' 37.56" N, 88° 23' 43.08" E	2019	1263/CBT
144	AP-144	Gazole	West Bengal, Maldah	25° 10' 40.80" N, 88° 14' 45.60" E	2019	1258/CBT
145	AP-145	Asansol	West Bengal, Paschim Bardhaman	23° 40' 22.80" N, 86° 57' 07.20" E	2019	1260/CBT
146	AP-146	Bardhaman	West Bengal, Purba Bardhaman	23° 13' 55.20" N, 87° 51' 39.60" E	2019	1261/CBT
147	AP-147	chowbaga	West Bengal, kolkata	22° 31' 27.23" N, 88° 25' 02.48" E	2019	1243/CBT
148	AP-148	Jadavpur	West Bengal, Kolkata	22° 29' 43.80" N, 88° 22' 15.24" E	2019	1244/CBT
149	AP-149	Saktipur	West Bengal, Murshidabad	24° 08′ 24.00″ N, 88° 15′ 36.00″ E	2019	1259/CBT
150	AP-150	Kashipur	Purulia, WB	23° 26' 02.40" N, 86° 40' 01.20" E	2019	1249/CBT

Table S5. Physicochemical properties of soil samples collected from different geographical locations of Odisha.

Serial No.	Acc. No.	pH	EC (Ds/m)	OC (%)	N (kg/ha)	P(kg/ha)	K(kg/ha)	S(kg/ha)
1	AP-1	5.9	0.2	0.25	120	20.18	170	47
2	AP-2	4.52	0.1	0.69	145	17.89	153	60
3	AP-3	4.65	0.09	0.74	103	15	167.1	43
4	AP-4	5.81	0.09	0.75	95	17.98	161.9	30.5
5	AP-5	4.53	0.08	0.73	34	39.78	385.2	60
6	AP-6	4.52	0.1	0.69	107	27	253	60

7	AP-7	4.65	0.09	0.74	33	40	607.1	43
8	AP-8	5.81	0.09	0.75	46	38.5	361.9	30.5
9	AP-9	5.83	0.31	0.57	98	24.5	232.5	49
10	AP-10	5.4	0.15	0.33	80	18.56	160.8	12.95
11	AP-11	5.5	0.06	0.34	76	19.99	90.45	7.55
12	AP-12	5.2	0.1	0.42	127	19.92	160.3	9.25
13	AP-13	5.8	0.35	0.3	64	27.43	167	105.95
14	AP-14	5.8	0.07	0.5	125	16.22	137	142
15	AP-15	6.1	0.11	0.5	114	21.77	181	56.25
16	AP-16	6.4	0.21	1	132	23.43	166	19.06
17	AP-17	4.7	0.18	0.4	182	9.76	203	17.33
18	AP-18	5.85	0.57	0.68	182	12.24	94	42.46
19	AP-19	6.12	0.49	0.41	164	10.79	76	18.44
20	AP-20	5.56	0.02	0.44	180	17	88	0.26
21	AP-21	5.42	0.25	0.5	102	11.7	104	126
22	AP-22	5.57	0.26	0.6	117	21.43	111	3.7
23	AP-23	5.91	0.02	0.59	68	28.45	261.9	25
24	AP-24	5.44	0.05	0.56	79	27.37	208.9	35
25	AP-25	5.85	0.07	0.58	38	39.33	445.9	41.25
26	AP-26	4.59	0.03	0.56	70	32.89	252.7	11.25
27	AP-27	5.91	0.02	0.49	68	34.5	270.9	25
28	AP-28	6.09	0.05	0.56	30	49	708.9	35
29	AP-29	5.98	0.01	0.87	30	43.29	653.9	41.25
30	AP-30	5.85	0.07	0.58	58	35.24	245.9	11.25
31	AP-31	5.85	0.07	0.58	70	35.24	245.9	32.5
32	AP-32	5.25	0.05	0.56	30	38.77	353.2	16.25
33	AP-33	5.44	0.05	0.55	75	33.12	290.2	16.25
34	AP-34	5.57	0.25	0.77	200	15.21	65	11.44
35	AP-35	4.87	0.32	0.44	210	16	55.4	88
36	AP-36	5.79	0.3	0.11	86	20.55	112.1	310
37	AP-37	5.72	0.07	0.47	49	37.912	338	11.12
38	AP-38	4.75	0.08	0.12	52	39.22	383	1670
39	AP-39	5.62	0.08	0.35	140	11.1	60	26
40	AP-40	6.07	0.15	0.35	44	46.11	384	1670
41	AP-41	5.8	0.15	0.2	145	16	164	1670
42	AP-42	5.27	0.1	0.4	95	25	201	27.5
43	AP-43	5.21	0.06	0.35	102	19.1	204	1670
44	AP-44	5.2	0.06	0.51	41	38.45	289	3.38
45	AP-45	4.8	0.09	0.19	82	26.88	269	1.84
46	AP-46	4.9	0.2	0.27	107	21.67	207	0.92

47	AP-47	4.6	0.01	0.47	76	29.78	251	3.99
48	AP-48	4.9	0.1	0.54	124	14.8	99	5.21
49	AP-49	4.8	0.3	0.69	150	15.89	162	1.84
50	AP-50	5.2	0.02	0.92	70	30.05	291	2.15
51	AP-51	6.1	0.01	0.51	77	33.4	332	0.31
52	AP-52	5.2	0.01	1.4	80	35.4	296	2.15
53	AP-53	7	0.01	0.73	68	33.2	225	4.6
54	AP-54	5.2	0.01	0.73	69	33.2	225	1.53
55	AP-55	4.52	0.08	0.32	90	28.43	198	15.93
56	AP-56	6.6	0.38	0.6	97	29.25	297	28.6
57	AP-57	5.9	0.53	0.78	83	25.5	220	17.12
58	AP-58	5.94	0.2	0.42	90	26.25	245	4.34
59	AP-59	5.6	0.12	0.41	95	28.75	232	28.96
60	AP-60	5.92	0.14	0.9	78	29	234	33.7
61	AP-61	5.42	0.06	0.4	118	12.5	167	28.96
62	AP-62	5.41	0.05	0.32	100	12	187	51.38
63	AP-63	5.23	0.25	0.24	87	11.5	428	6.52
64	AP-64	5.22	0.21	0.43	125	10.75	196	4.34
65	AP-65	5.56	0.26	0.18	64	20	298	15.2
66	AP-66	6.29	0.16	0.6	76	30	262	22.29
67	AP-67	5.13	0.18	0.19	50	29.25	444	38.01
68	AP-68	5.13	0.18	0.19	90	26.25	245	38.01
69	AP-69	5.2	0.52	0.32	87	27.75	307	23.26
70	AP-70	6.2	0.12	0.32	32	48.33	424	120
71	AP-71	5.7	0.2	0.48	74	25.1	302	480
72	AP-72	5.4	0.2	0.48	112	12	220	138
73	AP-73	5.07	0.42	0.25	156	17	90	37.5
74	AP-74	4.08	0.07	0.69	112	23.88	99.89	1.69
75	AP-75	5.76	0.33	0.39	178	7.2	87.9	2.78
76	AP-76	5.8	0.64	0.4	142	12	180	370
77	AP-77	5.6	0.56	0.54	70	16	289	10.2
78	AP-78	5.8	0.42	0.44	40	46.31	234	10.8
79	AP-79	6	0.33	0.37	136.4	24.15	213	8
80	AP-80	6.4	0.26	0.35	172.4	14.9	222	2
81	AP-81	7.7	0.44	0.63	65	38.7	233	5
82	AP-82	5.48	0.33	0.5	164	12.9	82	1.75
83	AP-83	7.14	0.36	1.1	90	28.99	199.1	13.65
84	AP-84	8.32	0.52	0.78	101	28.77	233	17.76
85	AP-85	5.42	0.39	1.01	150	16	203	17.38
86	AP-86	4.4	0.1	0.6	136	14.99	150	40.95

87	AP-87	5.1	0.1	0.64	143	16.78	170	30.54
88	AP-88	5.5	0.2	0.14	119	19.33	120	1.04
89	AP-89	5.86	0.17	0.29	147	14.32	189.26	16.25
90	AP-90	6.42	0.1	0.12	125	7.84	205.6	6.25
91	AP-91	5.03	0.26	0.34	160	14.56	66.32	5
92	AP-92	6.5	0.89	0.96	165	15.1	67.5	15
93	AP-93	5.9	1.2	0.65	230	13.89	147	9.04
94	AP-94	5.2	1.8	0.55	226	9.8	43.2	38.86
95	AP-95	6.32	0.73	0.3	180	19.91	156	5
96	AP-96	5.25	0.2	0.57	218	16.01	86	11.62
97	AP-97	5.9	1.2	0.65	231	11.5	30	9.04
98	AP-98	5.21	0.62	0.38	225	10	29	12
99	AP-99	5.95	1	0.98	97	28	135	6.12
100	AP-100	5.1	1.5	0.57	243	8.11	118	20.78
101	AP-101	5.9	0.59	0.84	230	8.99	126	10.35
102	AP-102	5.93	0.79	0.69	135	29	178	28.08
103	AP-103	6.5	0.89	0.96	192	31	67.5	15
104	AP-104	6.92	0.97	0.99	189	21	42	7.39
105	AP-105	6.23	1.9	0.53	183	28.4	86	10.77
106	AP-106	5.1	1.8	0.63	179	26	57	9.42
107	AP-107	6.7	1.6	0.65	185	30	134	23.27
108	AP-108	6.48	0.56	0.68	239	15.01	40	5.28
109	AP-109	5.5	2.6	0.41	134	23	137	11.7
110	AP-110	5.81	1	0.73	181	26	178	6.12
111	AP-111	6.2	0.76	0.76	178	23	263	5
112	AP-112	6.51	2.9	0.62	155	29	218	10.98
113	AP-113	7	0.23	0.27	170	12.36	54	2.8
114	AP-114	6.9	0.09	0.37	243	13.56	56	2.1
115	AP-115	7	0.23	0.27	227	12.8	45	2.8
116	AP-116	6.9	0.09	0.37	222.4	13	67	2.1
117	AP-117	6.8	0.09	0.35	219.4	16.09	230	2.2
118	AP-118	6.2	0.42	0.78	230	14.3	70	30
119	AP-119	6.35	0.41	0.38	173	32	185	12.4
120	AP-120	6.9	0.09	0.37	157	30	228	2.1
121	AP-121	6.9	0.09	0.37	171	32	228	2.1
122	AP-122	5.23	0.98	0.63	149	30	206	23.65
123	AP-123	5.69	0.11	0.57	159	15	128	13.63
124	AP-124	5.88	0.17	0.59	210	17	184	10.93
125	AP-125	5.84	0.06	0.81	123	25	180	11.7
126	AP-126	4.45	0.19	0.93	97	27	167	15.64

127	AP-127	6.9	0.06	0.36	170	14.1	161	1.8
128	AP-128	4.91	0.61	0.62	247	7	30	12.7
129	AP-129	7.82	0.17	0.72	106	27	334.6	20.6
130	AP-130	4.95	0.11	0.63	217	16	136	11.35
131	AP-131	7.2	0.29	0.42	201	18	109	7.81
132	AP-132	5.7	0.69	0.42	218	17	110	2.96
133	AP-133	6.9	1.3	0.47	65	41	140	5.7
134	AP-134	5.9	0.39	0.42	145	35	268	6.12
135	AP-135	5.2	0.14	0.78	235	8.13	35	6
136	AP-136	5.4	0.15	0.55	232	9.1	46	6
137	AP-137	5.2	0.31	0.81	155	15	234	4
138	AP-138	5.9	0.31	0.88	230	17	78	4
139	AP-139	5.32	0.27	0.46	150	18	253	14.46
140	AP-140	7.57	0.44	0.53	189	20.1	292	18.56
141	AP-141	8.37	0.1	0.48	175	25	156	12.33
142	AP-142	6.35	0.41	0.38	129	26	185	12.4
143	AP-143	5.44	0.01	1.08	241	12.78	120	8.59
144	AP-144	4.7	0.21	0.15	170	24	188	4.6
145	AP-145	5.3	0.17	0.54	195	23	175	9
146	AP-146	4.8	0.05	0.49	107	15	189	13.59
147	AP-147	5.7	0.24	1.1	235	10	54	8
148	AP-148	5.6	0.36	1.2	99	34	245	14
149	AP-149	6.9	0.17	0.67	229	15	50	4.2
150	AP-150	6.4	0.43	0.3	140	35	215	4

Table S6. Climatic data for *Andrographis paniculata* accessions from different geographical regions of Eastern India.

Serial No.	Acc. No.	Altitude	Annual Precipitation(mm)	Max temp.(°C)	Minimum temp.(°C)	Average temp.(°C)	Annual RH	UV Index
1	AP-1	47	1505	36.6	15.6	27.4	78.79	6.67
2	AP-2	39	1428	36.1	15.7	27.2	67.50	6.67
3	AP-3	43	1428	36.1	15.7	27.2	67.50	6.67
4	AP-4	39	1428	36.1	15.7	27.2	67.50	6.67
5	AP-5	39	1428	36.1	15.7	27.2	67.50	6.67
6	AP-6	43	1428	36.1	15.7	27.2	67.50	6.67
7	AP-7	43	1428	36.1	15.7	27.2	67.50	6.67
8	AP-8	43	1428	36.1	15.7	27.2	67.50	6.67
9	AP-9	43	1428	36.1	15.7	27.2	67.50	6.67
10	AP-10	6	1486	36.2	14	26.8	76.14	7.08

11	AP-11	53	1598	36.2	14	26.7	76.14	7.08
12	AP-12	19	1496	36.2	14	26.9	76.14	7.08
13	AP-13	20	1191	32.5	16.6	26.7	63.50	6.83
14	AP-14	21	1194	32.5	16.6	26.8	63.50	6.83
15	AP-15	16	1194	32.5	16.6	26.8	63.50	6.83
16	AP-16	46	1194	33.4	13.7	26.8	63.50	6.83
17	AP-17	46	1194	33.4	13.7	26.8	63.50	6.83
18	AP-18	95	1194	32.5	16.6	26.8	63.50	6.83
19	AP-19	188	1194	32.5	16.6	26.8	63.50	6.83
20	AP-20	29	1515	38.3	15.5	27.6	80.36	6.83
21	AP-21	50	1515	38.3	15.5	27.6	65.92	6.83
22	AP-22	29	1515	38.3	15.5	27.6	65.92	6.83
23	AP-23	59	1248	33.4	13.7	24.8	67.00	6.75
24	AP-24	1089	1247	33.4	13.7	24.7	67.00	6.75
25	AP-25	658	1245	33.4	13.7	24.8	67.00	6.75
26	AP-26	124	1249	33.4	13.7	24.8	67.00	6.75
27	AP-27	167	1248	33.4	13.7	24.8	67.00	6.75
28	AP-28	124	1247	33.4	13.7	24.7	67.00	6.75
29	AP-29	124	1245	33.4	13.7	24.8	67.00	6.75
30	AP-30	658	1249	33.4	13.7	24.8	67.00	6.75
31	AP-31	658	1249	33.4	13.7	24.8	67.00	6.75
32	AP-32	429	1249	33.4	13.7	24.8	67.00	6.75
33	AP-33	71	1249	33.4	13.7	24.8	67.00	6.75
34	AP-34	11	1428	34.9	16.4	27.2	61.17	6.67
35	AP-35	9	1428	34.9	16.4	27.3	61.17	6.67
36	AP-36	9	1428	34.9	16.4	27.4	61.17	6.67
37	AP-37	13	1428	34.9	16.4	27.5	63.50	6.75
38	AP-38	57	1472	37.9	14.6	27	61.00	6.83
39	AP-39	116	1472	37.9	14.6	27	61.00	6.83
40	AP-40	124	1472	37.9	14.6	27	65.92	6.83
41	AP-41	187	1472	37.9	14.6	27	65.92	6.83
42	AP-42	116	1472	37.9	14.6	27	65.92	6.83
43	AP-43	240	1472	37.9	14.6	27	65.92	6.83
44	AP-44	33	1465	37.2	13.7	27.3	59.25	7.08
45	AP-45	40	1610	38.5	14.2	26.1	59.25	7.08
46	AP-46	27	1459	38.5	14.2	27.1	59.25	7.08
47	AP-47	40	1670	37.2	13.7	26.7	59.25	7.08
48	AP-48	388	1610	38.5	14.2	26.1	59.25	7.08
49	AP-49	403	1459	38.5	14.2	27.1	59.25	7.08
50	AP-50	573	1459	38.5	14.2	27.1	59.25	7.08

51	AP-51	573	1308	38.5	14.2	26.1	59.25	7.08
52	AP-52	573	1157	38.5	14.2	27	59.25	7.08
53	AP-53	325	855	38.5	14.2	27.3	59.25	7.08
54	AP-54	114	704	38.5	14.2	26.1	59.25	7.08
55	AP-55	93	1516	37.3	14.5	26.9	65.42	6.67
56	AP-56	138	1516	37.3	14.5	26.9	65.42	6.67
57	AP-57	121	1516	37.3	14.5	26.9	65.42	6.67
58	AP-58	69	1516	37.3	14.5	26.9	65.42	6.67
59	AP-59	91	1516	37.3	14.5	26.9	65.42	6.67
60	AP-60	87	1516	37.3	14.5	26.9	65.42	6.67
61	AP-61	88	1396	38.2	14.7	27.2	65.42	6.67
62	AP-62	43	1396	38.2	14.7	27.2	65.42	6.67
63	AP-63	128	1396	38.2	14.7	27.2	65.42	6.67
64	AP-64	69	1396	38.2	14.7	27.2	65.42	6.67
65	AP-65	101	1396	38.2	14.7	27.2	65.42	6.67
66	AP-66	247	1396	38.2	14.7	27.2	65.42	6.67
67	AP-67	69	1396	38.2	14.7	27.2	65.42	6.67
68	AP-68	93	1396	38.2	14.7	27.2	65.42	6.67
69	AP-69	64	1396	38.2	14.7	27.2	65.42	6.67
70	AP-70	755	1323	37.5	12.1	25.1	55.50	6.83
71	AP-71	740	1323	37.5	12.1	25.1	55.50	6.83
72	AP-72	722	1323	37.5	12.1	25.1	55.50	6.83
73	AP-73	28	1516	37.3	14.5	26.9	62.42	7.08
74	AP-74	19	1516	37.3	14.5	26.9	62.42	7.08
75	AP-75	14	1516	37.3	14.5	26.9	62.42	7.08
76	AP-76	541	1323	37.5	12.1	25.1	55.50	6.83
77	AP-77	369	1253	39.4	13.6	26.7	55.50	6.75
78	AP-78	342	1253	39.4	13.6	26.7	55.42	6.75
79	AP-79	471	1494	35.7	15.2	26.9	64.25	6.33
80	AP-80	511	1494	35.7	15.2	26.9	64.25	6.33
81	AP-81	56	1494	35.7	15.2	26.9	64.25	6.33
82	AP-82	15	1515	38.3	15.5	27.6	67.25	6.67
83	AP-83	204	1249	40	13.4	26.9	57.92	6.83
84	AP-84	114	1249	40	13.4	26.9	57.92	6.83
85	AP-85	257	1249	40	13.4	26.9	57.92	6.83
86	AP-86	155	1638	42.1	12.2	26.8	55.25	6.83
87	AP-87	188	1638	42.1	12.2	26.8	55.25	6.83
88	AP-88	163	1638	42.1	12.2	26.8	55.25	6.83
89	AP-89	249	1312	36.7	14.9	26.5	68.58	6.58
90	AP-90	223	1312	36.7	14.9	26.5	68.58	6.58

91	AP-91	121	1312	36.7	14.9	26.5	68.58	6.58
92	AP-92	8	1735	35.9	12.6	26.2	57.83	6.83
93	AP-93	5	1333	37.8	11.4	26.4	63.08	6.92
94	AP-94	4	1399	34.6	13.7	26.3	62.92	6.83
95	AP-95	4	1472	34.1	14	26.3	63.25	6.90
96	AP-96	5	1333	37.8	11.4	26.4	63.08	6.92
97	AP-97	5	1333	37.8	11.4	26.4	63.08	6.92
98	AP-98	4	1657	33.2	14.7	26.3	58.00	6.83
99	AP-99	6	1157	38.4	12.7	26.6	58.50	6.83
100	AP-100	4	1333	37.8	11.4	26.4	63.08	6.92
101	AP-101	8	1609	35.1	13.1	26.3	61.00	6.83
102	AP-102	4	1592	33.5	14.3	26.2	60.83	6.75
103	AP-103	8	1735	35.9	12.6	26.2	57.83	6.83
104	AP-104	6	1607	34.8	13.2	26.3	56.50	6.75
105	AP-105	4	1333	37.8	11.4	26.4	63.08	6.92
106	AP-106	6	1472	34.1	14	26.3	63.25	6.90
107	AP-107	5	1399	34.6	13.7	26.3	62.92	6.83
108	AP-108	10	1609	35.1	13.1	26.3	61.00	6.83
109	AP-109	5	1657	33.2	14.7	26.3	58.00	6.83
110	AP-110	4	1657	33.2	14.7	26.3	58.00	6.83
111	AP-111	8	1233	38.2	12.2	26.4	53.42	7.25
112	AP-112	10	1609	35.1	13.1	26.4	61.00	6.83
113	AP-113	10	1533	36.4	12.3	26.4	59.42	6.92
114	AP-114	9	1569	36.4	12.4	26.4	59.42	6.92
115	AP-115	11	1527	36.2	12.3	26.3	58.83	6.75
116	AP-116	6	1689	35.9	12.6	26.3	59.33	6.67
117	AP-117	9	1533	36.4	12.3	26.4	59.42	6.92
118	AP-118	9	1627	36.3	12.4	26.4	59.33	6.92
119	AP-119	7	1535	36.3	11.7	26.2	60.08	7.00
120	AP-120	5	1574	35.8	11.8	26.2	60.67	6.92
121	AP-121	8	1527	36.2	12.3	26.3	58.83	6.75
122	AP-122	14	1533	36.4	12.3	26.4	59.42	6.92
123	AP-123	62	1311	38.1	11.8	26.4	55.50	7.17
124	AP-124	51	1289	38.3	11.8	26.3	55.67	7.33
125	AP-125	70	1307	38.2	11.4	26.2	54.00	7.34
126	AP-126	54	1287	38	12	26.3	55.33	7.08
127	AP-127	39	3197	32.2	10.2	24	59.83	6.67
128	AP-128	9	1260	38.7	12.7	26.8	57.50	6.92
129	AP-129	9	1533	36.4	12.3	26.4	59.42	6.92
130	AP-130	38	1306	38.3	13.1	26.8	57.08	7.08

131	AP-131	5	1580	34.2	14.5	26.6	67.00	6.83
132	AP-132	3	1550	35.6	13.6	26.5	61.00	7.08
133	AP-133	7	1451	34	14.1	26.4	63.08	6.92
134	AP-134	7	1526	34.5	14.5	26.5	59.42	6.92
135	AP-135	88	1236	38.6	12.5	26.6	53.42	7.25
136	AP-136	94	1236	38.6	12.5	26.6	53.42	7.25
137	AP-137	88	1236	38.6	12.5	26.6	53.42	7.25
138	AP-138	133	1193	38.5	12.4	26.5	53.42	7.17
139	AP-139	13	1299	37.8	11.5	26.4	58.92	7.17
140	AP-140	12	1345	36.8	11.9	26.3	60.25	7.08
141	AP-141	16	1350	37.8	11.6	26.4	57.58	7.08
142	AP-142	14	1353	37.7	11.2	26.4	58.92	7.25
143	AP-143	123	3274	32.5	9.7	24	61.17	6.00
144	AP-144	38	1442	35.6	10.5	25.2	56.75	6.83
145	AP-145	132	1298	38.9	12.1	26.5	50.83	7.25
146	AP-146	38	1313	37.6	12.6	26.3	57.92	7.17
147	AP-147	3	1735	35.9	12.6	26.2	57.92	6.83
148	AP-148	10	1607	34.8	13.2	26.3	56.50	6.75
149	AP-149	5	1365	37.7	11.7	26.3	56.17	7.08
150	AP-150	159	1164	39.4	12.3	26.5	49.42	7.25

RH=Relative humidity
